# The challenge of comorbidities: should we focus on clinical decision support as much as medical education?

**DOI:** 10.15694/mep.2021.000051.1

**Published:** 2021-02-22

**Authors:** Kieran Walsh

**Affiliations:** 1 BMJ

**Keywords:** Comorbidities, clinical decision support, medical education

## Abstract

This article was migrated. The article was marked as recommended.

Comorbidities present a great challenge to healthcare professionals and the health service. One in four patients now have two or more conditions. The prevalence of comorbidities increases with age and the population is continuing to grow older. Our response thus far has been slow and the solutions that have been suggested may not be scalable. There have been multiple calls for medical education to do more to address the issue of comorbidities. However existing ways of tackling this problem are unlikely to be scalable. It is impossible to learn and remember how to manage the many different potential combinations of acute diseases and comorbidities. Online clinical decision support has until the present enabled the management of patients with single conditions. But advances in clinical decision support can now enable the management of patients with multiple conditions.

## Clinical decision support for comorbidities

Comorbidities present a great challenge to healthcare professionals and the health service. One in four patients now have two or more conditions ( Barnett *et al.*, 2012). The prevalence of comorbidities increases with age and the population is continuing to grow older. Importantly comorbidities are also associated with socioeconomic deprivation ( Barnett *et al.*, 2012). And socioeconomically deprivation increases the likelihood that patients will develop comorbidities younger in life ( Whitty *et al.*, 2020). Mental health disorders are also associated with increased numbers of comorbid conditions ( Barnett *et al.*, 2012). Remarkably one in three emergency patients admitted to hospital in the UK have five or more conditions ( Steventon *et al.*, 2018). Patients with comorbidities spend longer in hospital and this increased length of stay is associated with increased costs ( Steventon *et al.*, 2018). The challenge of comorbidities has traditionally been seen as a problem for high income countries, but the problem is now increasing in middle income countries also ( Whitty *et al.*, 2020). Comorbidities represent a problem for health systems - and also for individual patients. There are multiple examples. Just one example is that of COPD and type 2 diabetes - both very common conditions. Up to twenty percent of patients with COPD also have type 2 diabetes ( Parappil *et al.*, 2010). Yet the treatment for one condition can make the other one worse - for example steroids for exacerbations of COPD can cause hyperglycemia. At a patient, professional and population level, comorbidities present a massive challenge to the health service.

Our response thus far has been slow and the solutions that have been suggested may not be scalable. There have been multiple calls for medical education to do more to address the issue of comorbidities ( Whitty *et al.*, 2020). Whitty *et al.* have written that “training from medical school onwards, clinical teams, and clinical guidelines, however, all tend to be organised along single disease or single organ lines.” ( Whitty *et al.*, 2020). The authors call for the “management of coexisting physical and mental health problems” to be “embedded into medical training and continuous professional development, including for specialists.” ( Whitty *et al.*, 2020). There are multiple recommendations on how to educate healthcare professionals in the management of patients with comorbidities. Guidance from the National Institute for Health and Care Excellence (NICE) in the UK contains generic suggestions that would be relevant to all patients with comorbidities. These include “knowing the patient as an individual, tailoring healthcare services for each patient, continuity of care and relationships, and enabling patients to actively participate in their care.” ( Nice, 2016) It suggests communication and team working skills that will enable this to happen. It also advises that healthcare professionals should: “after a discussion of disease and treatment burden and the person’s, personal goals, values and priorities, develop and agree an individualised management plan with the person.” ( Nice, 2016)

This is helpful - but only up to a point. Communication skills and team working skills are important for patients with multiple illnesses. And the patient should be seen as the most important member of the team. However these skills are equally important in the management of patients with single conditions. And a definite shortcoming is that generic skills will not give the healthcare professional everything that they need to know to answer the patient’s questions. Ultimately a patient with the common combination of COPD and diabetes will want to know what the best way is to treat these conditions. Their doctor will need to be able to tell them. And they will need to be confident that they are giving an evidence-based answer and that their recommendations for the treatment of one condition will not make the other condition worse. They will also need to be reassured that treatment for one condition will not lead to other conditions being neglected or overlooked.

There are few resources that can help with this. There are occasional educational articles that outline how to manage a patient with more than one illness ( Stamp and Chapman, 2013; Neder *et al.*, 2018). But these are hard to find and harder to quality assure. In any case, this approach is not scalable - simply due the exponential number of potential combinations of comorbidities. For example a patient with COPD might have diabetes, or hypertension, or diabetes and hypertension. This is a common scenario - with very many patients in hospital having very many comorbidities ( Steventon *et al.*, 2018). Even looking up just two different guidelines is impossible in the acute setting - the shortest guidelines are at least several pages long. There is no time for busy healthcare professionals to do this. It is certainly impossible to learn and remember how to manage the many different potential combinations of acute diseases and comorbidities.

It is tempting to think that artificial intelligence might have the answer. It might have an answer in the future but currently there are many barriers. These include the unintended consequences of artificial intelligence, its complete dependence on data (which is not always reliable), and the potential of artificial intelligence to discriminate against certain groups of patients or learners ( Walsh, 2020). This last issue is especially important in the context of patients with comorbidities - who often come from socially deprived sections of the population.

Thus artificial intelligence is unlikely to be the solution to this problem - as least not yet. But technology must be part of the solution - even if it does not involve machine learning but rather straightforward rule-based systems.

Online clinical decision support has until now enabled the management of patients with single conditions. But advances in clinical decision support can now enable the management of patients with multiple conditions.

The Comorbidities tool from BMJ Best Practice is built on functionality that enables healthcare professionals to add one or more comorbidities to an existing management plan and thus receive a holistic management plan. It has recently been launched and is in the process of being further developed and rolled out. It is relevant as a point of care clinical decision support tool but also as an educational tool. The reason is that healthcare professionals who use a comorbidities based clinical decision support tool are learning as they practice medicine. The reason why they use the tool is because they need to know something and thus have a learning need. They then engage in a sixty second bite of micro learning - a short but effective episode of learning. Finally they put their learning into action for the benefit of their patients. The whole process takes minutes, but it is based on sound educational principles - those of needs assessment, learning and putting learning into practice. If done properly with due attention to reflection on thoughts, actions and feelings all put into context, it is essentially self-regulated learning in action ( White, Gruppen and Fantone, 2014).

Context is vitally important and, in today’s world, multiple overlapping and complex comorbidities are now part of the contextualised norm. This new world has been brought into stark relief by the Covid-19 pandemic. Espinosa and colleagues conducted a meta-analysis of comorbidities in patients with Covid-19 ( Espinosa *et al.*, 2020). They found that 42% of patients with Covid-19 had comorbidities; 61% of those admitted to the Intensive Care Unit had comorbidities; and 77% of those who died had comorbidities. Hypertension was the most prevalent comorbidity (affecting 32% of patients). Other common comorbidities included diabetes (22%), heart disease (13%), and COPD (8%). It is patients with comorbidities who are becoming seriously ill.

Tackling the issue of comorbidities will be essential in managing the Covid-19 pandemic. But comorbidities will remain a challenge for health services far into the future. Medical education that is focussed on comorbidities will help. But it won’t be enough on its own. Advanced forms of clinical decision support will be essential to the better management of patients with multiple illnesses.

## Take Home Messages

• Comorbidities present a great challenge to healthcare professionals and the health service.
• There have been multiple calls for medical education to do more to address the issue of comorbidities.
• Existing ways of tackling this problem are unlikely to be scalable.
• Advances in clinical decision support can now enable the management of patients with multiple conditions.

## Notes On Contributors

**Dr Kieran Walsh** is Clinical Director at BMJ. He is the clinical lead of the medical education and clinical decision support resources at BMJ. He has a vast amount of experience in online medical education, clinical decision support, face to face delivery of medical education, and summative and formative assessment.

## Declarations

The author has declared the conflicts of interest below.

Kieran Walsh works for BMJ which produces a range of resources in medical education and clinical decision support.

## Ethics Statement

This is a personal view and not research.

## External Funding

This article has not had any External Funding
