# Supplementary material for: The challenge of comorbidities: should we focus on clinical decision support as much as medical education?
Source: MedEdPublish (2016). 2021 Feb 22;10:51. [Version 1] doi: 10.15694/mep.2021.000051.1 (PMC10939611; doi:10.15694/mep.2021.000051.1)
Supplement: Peer review history

# Reviewer report: Reviewer response for version 1

**DOI:** 10.21956/mep.18884.r26956

**Published:** 2021-03-06

**Authors:** Megan Anakin

**Affiliations:** 1 University of Otago

This review has been migrated. The reviewer awarded 2 stars out of 5 The topic of comorbidities caught my eye because it is an important area in health professions education. This opinion piece focuses on arguing that decision support software may be useful in managing complexity and that educators should consider how we use it in medical education. My comments are intended to assist the author to extend this brief article and enhance the argument presented with further description and details. Please consider revising the title because the implied comparison between decision support and medical education does not clearly express that the comparison presented is about current methods used to teach and learn about managing comorbidities and a new method described in the article. The claim that learning to manage comorbidities is “impossible” is an absolute statement, therefore, it needs a supporting reference, otherwise it should be rephrased with ‘hedging’ language to show that that in education there are always choices. Further explanation should be provided to explain why “Our response thus far has been slow and the solutions that have been suggested may not be scalable” and “Thus artificial intelligence is unlikely to be the solution to this problem – as least not yet”. More than one example is needed to support the claim that “There are multiple recommendations on how to educate healthcare professionals in the management of patients with comorbidities”.References are needed to support the factual claims made in the paragraph in the ‘Clinical decision support for comorbidities’. Please consider combining the last two paragraphs of the article so the relevance of managing comorbidities in the COVID19 era is more strongly connected to the need to provide enhance our teach and learn methods about managing comorbidities. Please consider revising the sentence: “But it won’t be enough on its own” to remove the contraction and specify the subject to make the sentence more formal and complete.I would be very happy to read and review a revised version of this article.

Reviewer Expertise:

NA

No decision status is available

# Reviewer report: Reviewer response for version 1

**DOI:** 10.21956/mep.18884.r26958

**Published:** 2021-02-25

**Authors:** Balakrishnan Nair

**Affiliations:** 1 Centre for Medical Professional Development and University of Newcastle

This review has been migrated. The reviewer awarded 5 stars out of 5 This is a timely article. With the increasing ageing of the population, co-morbidities are on the rise. It is associated with polypharmacy. With the growing specialisation and single organ doctors , patients see too many specialists who do not communicate with each other leading to frustrated and confused patients. We had looked at the PBL cases in medical schools and most of the cases are single organ problems which are sorted in a very short time frame, a far cry from real life. We have evidence-based medicine for single diseases; what we need is Medicine -based evidence. So if we need to prepare our future doctors , we need to teach them about co-morbidities and how to care for their patients

Reviewer Expertise:

NA

No decision status is available

# Reviewer report: Reviewer response for version 1

**DOI:** 10.21956/mep.18884.r26957

**Published:** 2021-02-23

**Authors:** BALAJI ARUMUGAM

**Affiliations:** 1 TAGORE MEDICAL COLLEGE AND HOSPITAL

This review has been migrated. The reviewer awarded 5 stars out of 5 Dear Dr Kieran Walsh is Clinical Director at BMJ,Thank you so much for your personal opinion article which is the most needed for all the medical practitioners globally to focus more on comorbidities in decision making for the treatment aspect of their own specialty. TO My PERSONAL OPINION Unfortunately, the overspecialization, compartmentalization of the mindset of the medical practitioners in managing the patients than treating them as a whole individual is becoming a problem for the patient as well as the treating specialist at some point of time. Due to co morbid illnesses - Patient side issues1. Increase in Number of visits 2. increase in number of drugs consumed (Often overlapped/overdose)3. Drug interactions and side effects4. Money and time spent The big solution would be as of now - Common Co morbid tool training in clinical general practice from the level of undergraduate itself with more comprehensive approach in postgraduate training in medical education to sensitize the medical professionals.

Reviewer Expertise:

NA

No decision status is available
